# Regulation of Neph3 gene in podocytes – key roles of transcription factors NF-κB and Sp1

**DOI:** 10.1186/1471-2199-10-83

**Published:** 2009-08-24

**Authors:** Mervi Ristola, Satu Arpiainen, Moin A Saleem, Peter W Mathieson, Gavin I Welsh, Sanna Lehtonen, Harry Holthöfer

**Affiliations:** 1 Department of Bacteriology and Immunology, Haartman Institute, P.O. Box 21, 00014 University of Helsinki, Finland; 2 National Centre for Sensor Research, Centre for BioAnalytical Sciences, Dublin City University, Glasnevin, Dublin 9, Ireland; 3 Academic and Children's Renal Unit, University of Bristol, Southmead Hospital, Bristol, BS10 5NB, UK

## Abstract

**Background:**

Neph3 (filtrin) is expressed in the glomerular podocytes where it localizes at the specialized cell adhesion structures of the foot processes called slit diaphragms which form the outermost layer of the glomerular filtration barrier. Neph3 protein shows homology and structural similarity to Neph1, Neph2 and nephrin, which all are crucial for maintaining the normal glomerular ultrafiltration function. The exact function of Neph3 in the kidney is not known but we have previously shown that the level of Neph3 mRNA is decreased in proteinuric diseases. This suggests that Neph3 may play a role in the pathogenesis of kidney damage, and emphasizes the need to analyze the regulatory mechanisms of Neph3 gene. In this study we investigated the transcriptional regulation of Neph3 gene by identifying transcription factors that control Neph3 expression.

**Results:**

We cloned and characterized approximately 5 kb fragment upstream of the Neph3 gene. Neph3 proximal promoter near the transcription start site was found to be devoid of TATA and CAAT boxes, but to contain a highly GC-rich area. Using promoter reporter gene constructs, we localized the main activating regulatory region of Neph3 gene in its proximal promoter region from -105 to -57. Within this region, putative transcription factor binding sites for NF-κB and Sp1 were found by computational analysis. Mutational screening indicated that NF-κB and Sp1 response elements are essential for the basal transcriptional activity of the Neph3 promoter. Co-transfection studies further showed that NF-κB and Sp1 regulate Neph3 promoter activity. In addition, overexpression of NF-κB increased endogenous Neph3 gene expression. Chromatin immunoprecipitation assay using cultured human podocytes demonstrated that both NF-κB and Sp1 interact with the Neph3 promoter.

**Conclusion:**

Our results show that NF-κB and Sp1 are key regulators of Neph3 expression at the basal level in podocytes, therefore providing new insight into the molecular mechanisms that contribute to the expression of Neph3 gene.

## Background

The glomerular filtration barrier consists of a fenestrated endothelium, a glomerular basement membrane and glomerular epithelial cells, podocytes. Podocytes surround the basement membrane of glomerular capillaries from the outside and present foot processes that are linked to each other with unique cell junction structures, the slit diaphragms (SD). According to the present view, SDs form the final barrier preventing leakage of plasma proteins from circulation to urine [1].

Neph3, also known as filtrin, is a member of the Neph (nephrin-like proteins) family and shows sequence homology and structural similarity to two other Neph proteins, Neph1 and Neph2, and to nephrin [2-5]. All these are transmembrane proteins that belong to the immunoglobulin superfamily [3-5]. In podocytes, Neph3, like other Neph family proteins and nephrin, localizes at the slit diaphragm [2,6-10]. Nephrin appears to be a key component of the SD and genetic nephrin deficiency results in the absence of SD and massive proteinuria in humans and mice [11-13]. Similarly, in Neph1-deficient mice, the podocyte foot processes are effaced and the mice exhibit severe proteinuria [14]. The function of Neph3 in the kidney is less well known but sequence homology and similar location with other Neph proteins and nephrin suggests that it has shared functions as a structural and signaling component of filtration barrier. In addition, the expression of Neph3 is down-regulated, similarly to nephrin mRNA, in human proteinuric diseases proposing it to have a role in maintaining normal SD structure and function [7]. However, very little is known about the mechanisms that regulate human Neph genes and the mechanisms behind the transcriptional regulation of Neph3 gene have not been elucidated at all.

To better understand the role of Neph3 in the SD under normal and pathophysiological conditions, we investigated the transcriptional regulation of Neph3 and identified the key regulatory regions in the Neph3 5' promoter. Further, we show that transcription factors nuclear factor-kappa B (NF-κB) and specificity protein 1 (Sp1) bind to the promoter and are essential in controlling Neph3 expression.

## Results

### Features of the upstream region of the human Neph3 gene

The human Neph3 gene (official HUGO gene name *KIRREL2*) consists of fifteen exons. It locates on chromosome 19q13.12, adjacent to nephrin, and encodes a 107 kDa protein. There are at least 5 different splicing variants of Neph3 that appear to have distinct tissue specificity [4,5]. All known variants have the same transcription start site. Mouse and rat have syntenic Neph3 gene regions in their chromosome locations 7qB1 and 1q21, respectively. We examined approximately 5000 bp 5' flanking region upstream from the Neph3 transcription start site [GenBank: AC002133]. Neph3 proximal promoter near the transcription start site was noticed to lack a typical TATA and CAAT boxes, but instead found to contain a highly GC-rich area. A CpG island (percentage of C or G: 63.1% and ratio of observed to expected CpG: 0.70) was located around the proximal promoter from -765 to +292 using the Blat tool . CpG islands are generally found in the promoter regions of housekeeping genes and the cytosines within these regions are typically kept unmethylated to allow the expression of the correlated genes [15]. The CpG island also includes several GC boxes, detected by MatInspector software (Genomatix), that are important for the expression of many different ubiquitous as well as tissue-specific cellular and viral genes [16]. Two primate specific Alu-repetition sequences were identified in the region -3021 to -2795 and from -2544 to -2317 bp upstream from the transcription start site using Genomic repeats tool in Genomatix.

### Location of the active promoter regions

**Figure 1:**
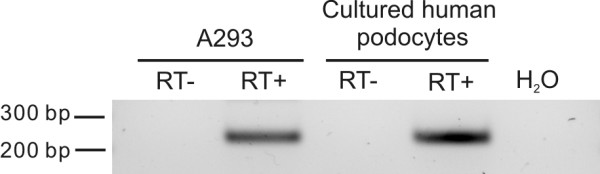
**Neph3 mRNA is expressed endogenously in human A293 embryonic kidney cell line and in differentiated human podocytes**. To determine the endogenous expression of Neph3 mRNA in A293 cells and differentiated human podocytes, total RNA was extracted, reverse transcribed, and expression of Neph3 mRNA was analyzed by conventional PCR using gene-specific primers. The amplification products were visualized on a 1.5% agarose gel. In the reverse transcriptase positive (RT+) samples, the amplification products were detectable and their identity was confirmed by sequencing. In the RT- samples and H_2_O sample, which represents a no-template control, no amplification products were detectable confirming the RNA origin of the PCR signals.

**Figure 2:**
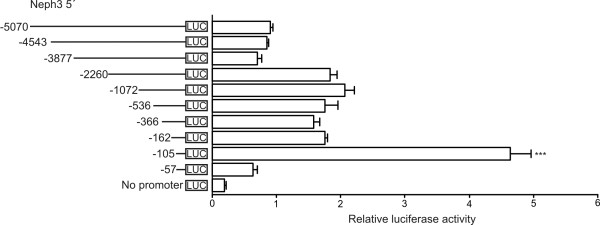
**Transcriptional activity of the 5'-flanking regions of the human Neph3 gene in A293 cells**. A series of luciferase (LUC) reporter plasmids containing various lengths of the Neph3 5'-flanking region was constructed. Negative numbers indicate the 5' end of the promoter fragment relative to the major transcription start site at +1. The reporter plasmids were transfected into A293 cells and luciferase activities were measured 24 h after transfection. The activities produced by the studied constructs were normalized against co-transfected control plasmid (phRL-TK) activities. The values represent means +S.D. of a representative experiment performed in triplicate. Each experiment was carried out three times with similar results. To confirm the results, statistical difference for the key observation (Neph3 5' -105 versus Neph3 5' -162) was calculated in three individual experiments using independent samples *t*-test. *** p < 0.001.

To locate the minimal sequence responsible for the basal transcription of the human Neph3 gene, a series of luciferase reporter plasmids containing various lengths of the Neph3 5'-flanking region were constructed and transiently transfected into human embryonic kidney cells (A293 cell line) that express Neph3 mRNA endogenously (Figure 1). As shown in Figure 2, already the Neph3 5' -57 promoter region activated the reporter gene to some extent, but the highest luciferase activity was produced by the Neph3 5' -105 construct. This indicates that the essential regulatory elements necessary for the Neph3 basal level transcription are located within the region of -105 to -57. The constructs Neph3 5' -2260, Neph3 5' -1072, Neph3 5' -536, Neph3 5' -366, Neph3 5' -162 showed approximately the same luciferase activity. Neph3 5' -3877 construct and Neph3 5' -162 construct had significantly lower luciferase activities compared to luciferase activities of Neph3 5' -2260 construct and Neph3 5' -105 construct, respectively, suggesting the presence of repressing transcription factor binding elements in the regions -3877 to -2260 and -162 to -105. Both identified Alu repetition sequences locate in the region from -3021 to -2317 in the Neph3 5' -3877 construct and may also be the reason for the lower activity of this part of the promoter. The longest construct Neph3 5' -5070 produced higher luciferase activity than Neph3 5' -4543 or Neph3 5' -3877 implying that in addition to proximal promoter region from -105 to -57, there is possibly also another, weaker enhancer site in the distal 5' region from -5070 to -3877.

### NF-κB and Sp1 response elements are essential for the basal transcriptional activity of the Neph3 promoter

**Figure 3:**
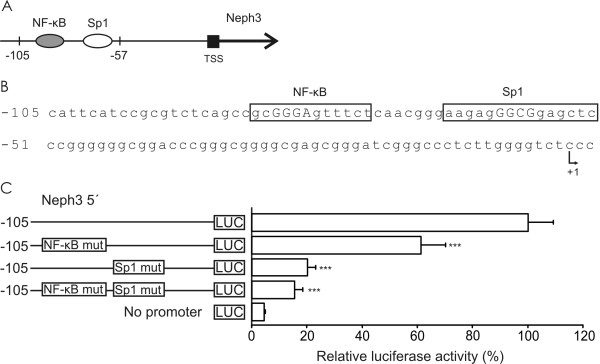
**NF-κB and Sp1 response elements are essential for the basal level transcription of Neph3 in A293 cells**. **(A) **A schematic representation of the Sp1 and NF-κB binding sites in the Neph3 promoter. **(B) **Nucleotide sequence of the human Neph3 promoter from -105 bp to TSS. The putative binding sites for NF-κB and Sp1 are boxed and core binding site is marked with Caps. The transcription start site is marked with +1. **(C) **Mutational analysis of the Sp1 and NF-κB binding sites in the Neph3 promoter. The Neph3 5' -Luc plasmids with mutated Sp1 and NF-κB binding sites were transfected into A293 cells and luciferase (LUC) activities were measured 24 h after transfection. The activities produced by the studied constructs were normalized against co-transfected control plasmid (phRL-TK) activities. The luciferase activities produced by the mutant constructs were compared with the activity produced by the unmutated construct. The values are presented as percentages of unmutated construct, which was set at 100% The values represent means +S.D. from three independent experiments performed in triplicate. Statistical difference of the promoter activity, using independent samples *t*-test, is shown against unmutated construct transfection. *** p < 0.001.

Reporter gene analysis indicated that the region of -105 to -57 in Neph3 promoter contains the essential regulatory elements. To locate putative binding sites for transcription factors within this promoter region, we used MatInspector software. The region was shown to contain putative binding sites for NF-κB (gcGGGAgtttct, where core binding site is marked with Caps) at the position -85 to -74 and for Sp1 (aagagGGCGgagctc) at the position -66 to -52 (Figures 3A and 3B). Core/matrix similarity values were 1.000/0.886 and 1.000/0.930 for the NF-κB and Sp1 binding sites, respectively. To investigate the functionality of these sites, NF-κB and Sp1 elements were mutated in the Neph3 5' -105 construct and the constructs were transfected into A293 cells. As shown in Figure 3C, mutation of NF-κB element decreased transcriptional activity by 39% and mutation of Sp1 element by even 80% indicating that both elements are functional and essential for the basal level transcription of Neph3. Double mutation of both elements decreased transcriptional activity of Neph3 5' -105 region to the same level as Sp1 binding site mutation alone.

### Overexpression of NF-κB and Sp1 activate Neph3 promoter

**Figure 4:**
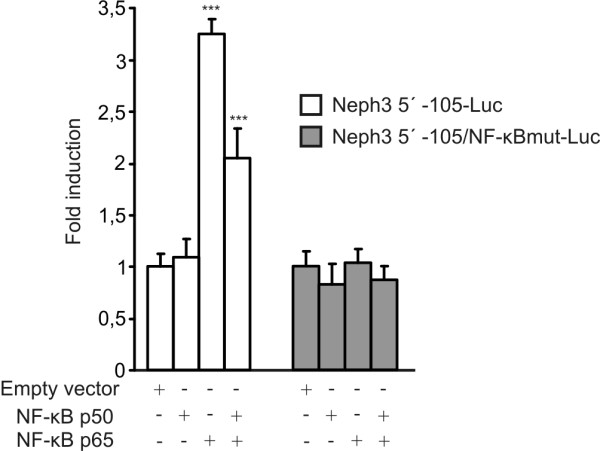
**Overexpression of NF-κB activates Neph3 promoter in A293 cells**. The expression vector for p50 or/and p65 NF-κB (NF-κB family members p50 and p65) was co-transfected with the Neph3 5' -105-Luc reporter plasmid or with reporter plasmid containing a mutated NF-κB binding site (Neph3 5' -105/NF-κBmut -Luc) into A293 cells and luciferase activities were measured 24 h after transfection. The activities produced by the studied constructs were normalized against co-transfected control plasmid (phRL-TK) activities. The results are presented as fold induction of the control experiment co-transfected with corresponding empty vector. The values represent means +S.D. from three independent experiments performed in triplicates. Statistical difference of the promoter activity, using independent samples *t*-test, is shown against the control co-transfection. *** p < 0.001.

Next we studied if NF-κB and Sp1 are able to activate Neph3 promoter. NF-κB is a transcription factor family consisting of different subunits which function as homo- or heterodimers. Heterodimer consisting of p50 and p65 subunits and p65 homodimer are the well known dimers participating in gene transactivation [17], and both p50 and p65 have been shown to be expressed in podocytes [18,19]. Therefore, we studied the effect of p50 and p65 overexpression on Neph3 promoter activity. Neph3 5' -105 construct was co-transfected together with NF-κB p50 and p65 expression vectors into A293 cells. As shown in Figure 4, p65 over-expression resulted in activation of Neph3 promoter with 3.3-fold induction compared to control. Over-expression of p50 subunit had no effect on Neph3 promoter activity. Simultaneous overexpression of p50 and p65 subunits led to no further activation of Neph3 promoter, but instead, to lower levels of activity than with p65 subunit alone. Furthermore, luciferase activity of Neph3 5' -105 construct containing mutated NF-κB binding site was not altered. These results suggested that NF-κB (subunit p65) is able to stimulate Neph3 transcription through NF-κB binding site in the position of -85 to -74.

**Figure 5:**
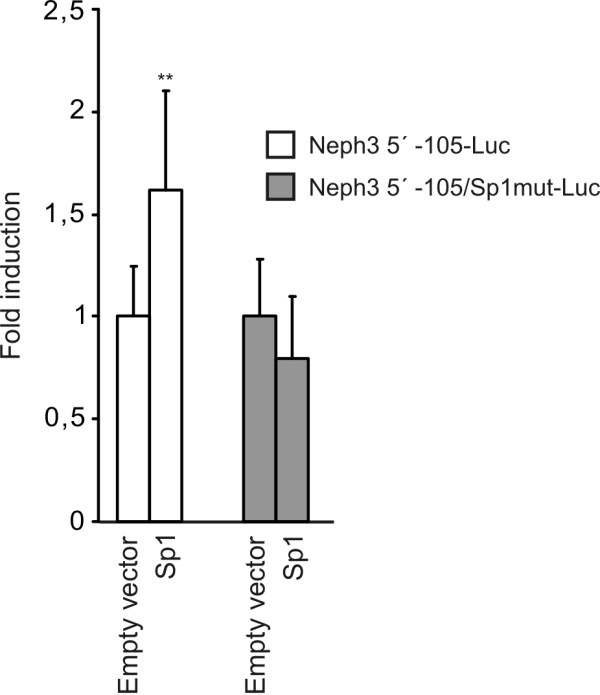
**Overexpression of Sp1 activates Neph3 promoter in *Drosophila *S2 cells**. The *Drosophila *expression vector for Sp1 was co-transfected with the Neph3 5' -105-Luc reporter plasmid or with reporter plasmid containing a mutated Sp1 binding site (Neph3 5' -105/Sp1mut-Luc) into *Drosophila *S2 cells and luciferase activities were measured 48 h after transfection. The activities produced by the studied constructs were normalized against protein concentration. The results are presented as fold induction of the control experiment co-transfected with corresponding empty vector. The values represent means +S.D. from three independent experiments performed in triplicates. Statistical difference of the promoter activity, using independent samples *t*-test, is shown against the control co-transfection. ** p < 0.01

Sp1 over-expression studies were carried out in *Drosophila *Schneider 2 (S2) cells. This is an established *in vitro *model to study the role of Sp1, since these cells lack endogenous Sp1, while all mammalian cell lines (including A293 cells) have high endogenous level of Sp1 and therefore are not suitable for Sp1 over-expression studies [20]. Neph3 5' -105 construct was co-transfected together with Sp1 expression vector, and as shown in Figure 5, Sp1 overexpression resulted in activation of Neph3 promoter with 1.6-fold induction compared to control. In contrast, mutation of Sp1 binding site abolished activation. These results indicated that Sp1 activates Neph3 transcription through Sp1 binding site in the position of -66 to -52.

### Overexpression of NF-κB increases Neph3 mRNA

**Figure 6:**
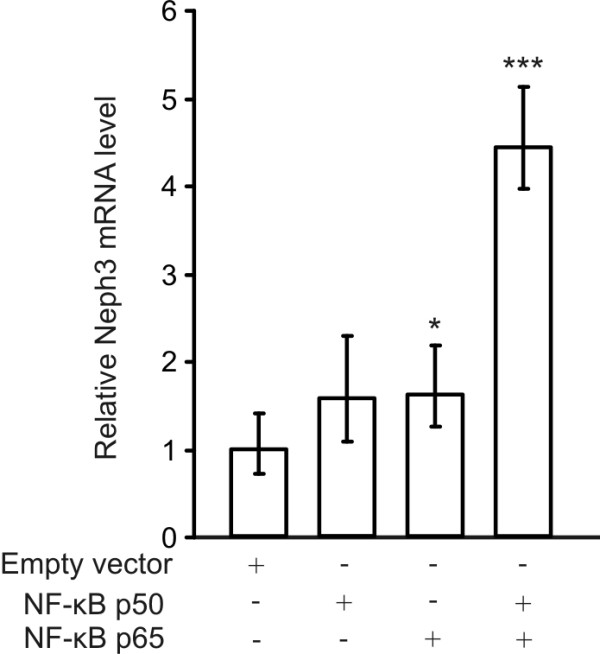
**Overexpression of NF-κB increases Neph3 mRNA in A293 cells**. A293 cells were transiently transfected with NF-κB p50 and p65 expression plasmids. After 48 h, cells were harvested and RNA was isolated. After reverse transcription, the Neph3 mRNA were quantified by quantitative reverse transcription-PCR and normalized to the GAPDH mRNA using the comparative C_t _method (ΔΔC_t_). The values represent means and range of three measurements from two individual experiments. Data is presented relative to the control transfection. The difference to the control transfection is statistically significant *** p < 0.001 or * p < 0.05 (independent samples *t*-test).

We also examined whether NF-κB has an effect on the expression of endogenous Neph3 mRNA. NF-κB p50 and p65 subunits were transiently transfected into A293 cells and after 48 h RNA was extracted and Neph3 mRNA was quantified by quantitative PCR. As shown in Figure 6, simultaneous over-expression of NF-κB p50 and p65 subunits increased Neph3 mRNA more than 4-fold compared to control. A lesser, although significant, increase of Neph3 mRNA was also seen when p65 subunit was transfected alone. In contrast, Neph3 mRNA level was not significantly different between p50 transfected and control cells. These findings demonstrate that forced expression of NF-κB p50 and p65 drive Neph3 gene expression in A293 cells.

### NF-κB and Sp1 bind to the promoter region of Neph3

**Figure 7:**
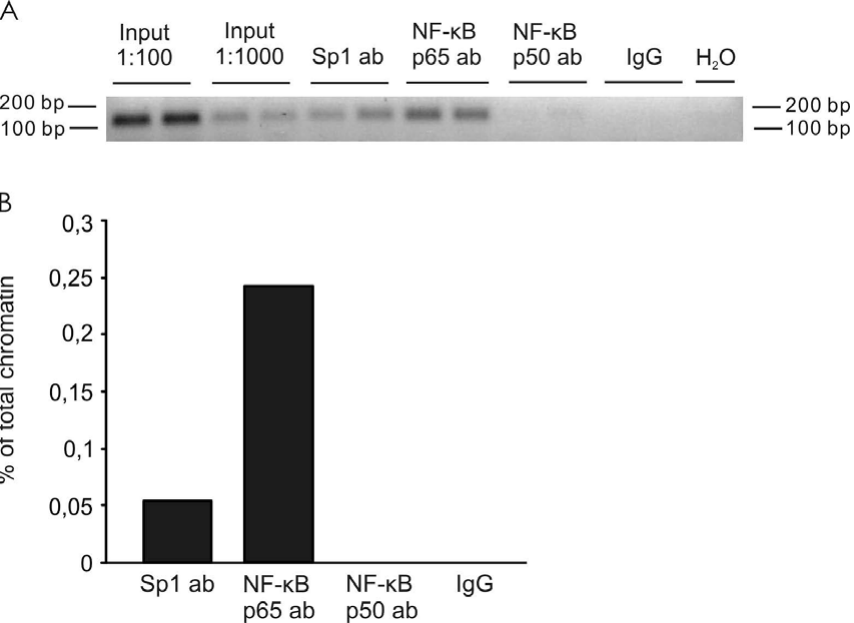
**NF-κB and Sp1 bind to the promoter region of Neph3 in cultured human podocytes**. In chromatin immunoprecipitation assays, NF-κB and Sp1 antibodies (ab) were used to precipitate DNA-protein complexes from differentiated human podocytes. Real-time PCR was performed with a specific primer pair (shown in Table 1) to amplify extracted DNA fragments. **(A) **Representative agarose gel: the input lanes confirm the successful PCR, the ab lanes detect the precipitated chromatin, rabbit IgG immunoprecipitation was used as a negative control and H_2_O sample represents a control sample that does not contain chromatin. **(B) **The relative quantity of DNA was counted by comparing the sample fluorescence to the fluorescence values measured from total chromatin input dilution series. Columns indicate the means of two parallel samples. The experiment was repeated twice with similar results.

In order to study the interaction of NF-κB and Sp1 with Neph3 promoter *ex vivo*, a chromatin immunoprecipitation assay was carried out. NF-κB p65, p50 and Sp1 antibodies were used to precipitate DNA-protein complexes from conditionally immortalized human podocyte cell line that express Neph3 mRNA endogenously (Figure 1). Immunoprecipitated DNA fragment including NF-κB and Sp1 binding sites was amplified by real-time PCR. Both NF-κB subunit p65 and Sp1 were shown to interact with Neph3 promoter, whereas NF-κB p50 antibody and rabbit IgG used as a negative control did not precipitate the promoter fragment (Figure 7). Due to different affinities of the used antibodies, the binding properties of the studied transcription factors cannot be compared.

## Discussion

Neph3 is a member of the nephrin-like protein family and localizes at the slit diaphragms of podocyte foot processes which form the outermost layer of the kidney filtration barrier. Besides the sequence homology of Neph3 with nephrin and other nephrin-like molecules, the known crucial components of the kidney filtration barrier, its structure, location, and down regulation in proteinuric diseases suggest that it plays an important role in maintaining the normal glomerular normal ultrafiltration. The aim of this study was to gain insight into the transcriptional regulation of Neph3 by identifying transcription factors that control Neph3 expression. Using reporter gene constructs, we identified the Neph3 5' promoter region that is essential for the basal level transcription. By computational analysis, the putative transcription factor binding elements for NF-κB and Sp1 in this region were identified within this region and shown to be functional by site-directed mutagenesis. Furthermore, over-expression and chromatin immunoprecipitation studies indicated that NF-κB and Sp1 indeed activate and bind to Neph3 promoter.

Sp1 is a member of Sp family of transcription factors which contain a conserved set of Cys_2_-His_2 _zinc-fingers representing a DNA-binding domain. Sp1 is ubiquitously expressed in mammalian cells. It can bind to and act through GC boxes to regulate gene expression of many housekeeping, tissue-specific, viral and inducible genes [21]. Previously, Sp1 has been shown to play a role in regulating basal level transcription of two other podocyte proteins, CD2-associated protein [22] and podocalyxin [23]. Sp1 is a constitutively active transcription factor [24], and Sp1 binding motifs are shown to be involved in the maintenance of the methylation-free status of the CpG islands [25]. Sp1 response element identified in this study also locates in the CpG island. Therefore, and similarly to the regulation of many other TATA-less genes [26], we propose that Sp1 functions as a key regulator of the Neph3 gene expression at the basal level.

NF-κB, a β-scaffold transcription factor family, plays an essential role in the regulation of many genes involved in immune and inflammatory responses, and in cell proliferation, differentiation and apoptosis [27]. The subcellular location of NF-κB is controlled by interactions with inhibitory proteins of the IκB family which in response to a large variety of stimuli, such as stress and cytokines, are phosphorylated, ubiquitinated and degraded thereby freeing NF-κB to translocate to the nucleus and to bind DNA at a response element of the consensus sequence 5'-GGGRNNYYCC-3' (R = A or G, N = any nucleotide, Y = C or T) [27]. NF-κB transcription factors exist as hetero- or homodimers and are formed by different subunits called p50, p52, c-Rel, RelA/p65 and RelB. Heterodimer consisting of p50 and p65 subunits and p65 homodimer are the well known dimers and the most abundant ones in most cell types, including podocytes [17-19,27] In our co-transfection experiments NF-κB p65 subunit together with Neph3 reporter gene construct resulted in a significant increase in Neph3 promoter activity. Similarly, the overexpression of p65 subunit increased endogenous Neph3 mRNA to some extend (p < 0.05). Interestingly, overexpression of p50 and p65 subunits together resulted in a high increase in the Neph3 mRNA level, but the results from the reporter gene assay did not show further increase in promoter activation by combination of p50 and p65. In addition, we could not detect p50 binding to the promoter in the ChIP assay. It might be that in the chromosomal context of the endogenous gene the p50 subunit binds the promoter and activates the Neph3 expression together with p65 although this is not seen with reporter gene constructs or fragmented chromosomes used in ChIP. However, based on these results it is difficult to conclude whether it is p65 homodimer or p50/p65 heterodimer that activates the transcription of the Neph3. Most likely, different NF-κB subunits may heterodimerize with p65 and regulate the expression of Neph3 in different ways under various (patho)physiological conditions.

We have previously shown that Neph3 mRNA is decreased in the kidneys of the diabetic nephropathy patients [7]. In line with this, nephrin mRNA has been shown to be downregulated in human diabetic nephropathy [28] and in addition, during the development of diabetic nephropathy in a rat model, nephrin mRNA is first transiently increased and later down-regulated [29]. Interestingly, the activation and nuclear translocation of NF-κB subunits p50 and p65 have been demonstrated in human diabetic nephropathy [30,31]. This, together with our results showing that NF-κB regulates Neph3 expression at the basal level (this study), prompts for further studies investigating whether NF-κB plays a role in the regulation of Neph3 (and possibly also other Neph family members and nephrin) under diabetic conditions.

The studies with renal biopsies from patients with acquired human glomerular diseases show correlation, besides in the mRNA expression of Neph3 and nephrin [7], also in the mRNA expression of nephrin and other podocyte-expressed genes, including synaptopodin and α-actinin-4 [32]. This suggests that common regulatory mechanisms may be activated in proteinuric glomerular diseases. Neph3 locates on the same chromosome as nephrin, 19q13.12, where these genes are arranged in a head-to-head orientation separated by a 5-kb region [5]. The genomic arrangement of Neph3 and nephrin together with their similar structure and location, in addition to the correlation in the mRNA expression of various podocyte-expressed genes [7,32] proposes that Neph3 and other podocyte genes may share key features in their regulation. However, whether NF-κB and Sp1 control the expression of nephrin or other podocyte genes is yet to be clarified.

Besides NF-κB and Sp1, additional transcription factors, both activating and inhibiting, are likely to participate in the regulation of Neph3. It has been shown that at least transcription factor Wilms tumor-1, Snail, retinoic acid receptors and peroxisome proliferator-activated receptors regulate nephrin [33-38], and could be involved also in Neph3 regulation. Interestingly, Sp1 has been demonstrated to interact with NF-κB, and cooperative functional and physical interactions between Sp1 and NF-κB have been shown to be required for example, for HIV-1 enhancer activation [39-41]. Our co-trasfection assays with Sp1 and NF-κB in A293 cells didn't show any activation of the Neph3 promoter co-operatively between Sp1 and NF-κB (data not shown). However, Sp1 overexpression studies are not feasible in mammalian cell lines because of their high endogenous level of Sp1, which may be near saturating with regard to induction of reporter gene expression. Regardless, it is possible that NF-κB and Sp1 function cooperatively in the Neph3 gene regulation at the endogenous level. In addition to kidney, Neph3 is also expressed in other tissues including the β-cells of the pancreas [4,42] and in the developing central nervous system in the brain where it is transiently expressed in early postmitotic neural precursor cells [43]. It is suggested that in pancreas and in brain Neph3 is involved in maintaining cell-cell contacts [4,43], but the exact function of Neph3 in these tissues has not yet been elucidated. Different splicing variants of Neph3 also appear to have distinct tissue specificity. Whether NF-κB and Sp1 also regulate Neph3 expression and the production of its different splicing variants in non-renal tissues remains to be elucidated.

## Conclusion

In conclusion, in this study we show that the main activating regulatory region of the Neph3 gene locates in its proximal promoter region between nucleotides -105 and -57. Further, we show that transcription factors Sp1 and NF-κB bind to this promoter region and are involved in the transcriptional regulation of the Neph3 gene at the basal level in podocytes, therefore providing new insight into the molecular mechanisms that contribute to the expression of Neph3 gene.

## Methods

### Cloning of a DNA segment between NPHS1 and Neph3 genes

Human genomic DNA was isolated from blood using AquaPure Genomic DNA Kit according to the manufacturer's protocol (Bio-Rad, Hercules, CA, USA). A 5276-bp DNA fragment between *NPHS1 *and Neph3 genes was amplified by PCR using DyNAzyme™ EXT DNA polymerase (Finnzymes, Espoo, Finland) from this genomic DNA using Neph3 5' -5070 genomic primer as sense primer and Neph3 5' +206 genomic primer as antisense primer (Table 1). The PCR product was subcloned into a pGlow-TOPO vector (Invitrogen, Carlsbad, CA, USA) and the presence of the insert was confirmed by restriction digestion and sequence analysis.

**Table 1:** Oligonucleotides used in the study

**Name**	**Position**^**a**^	**Sequence 5'-3'**
*Primers for endogenous Neph3 mRNA detection*		

Neph3 5' +481-FW endogenous	+481 to +499	TTAGGCCCGTGGAGCTAGA

Neph3 5' +702-RV endogenous	+702 to +683	CATCTCGGAACCACAGCAAT

*Primers for genomic DNA cloning*		

Neph3 5' -5070-FW genomic	-5070 to -5051	ATCGCGGATCCCACAGGTCCCCCTACTGTGA

Neph3 5' +206-RV genomic	+206 to +187	CCCAAGGTTCACGAGATTTG

*Primers for reporter constructs*		

Neph3 5' +48-RV	+48 to +31	GACAGATCTCTCTGACGCTCTGAAACG

Neph3 5' -5070-FW	-5070 to -5053	GACACGCGTCACAGGTCCCCCTACTGT

Neph3 5' -4543-FW	-4543 to -4523	GACACGCGTCCTTGTCTCACTACTCACAGC

Neph3 5' -3877-FW	-3877 to -3860	GACACGCGTGTGATCCATCTGCCTCAG

Neph3 5' -2260-FW	-2260 to -2240	GACACGCGTCTGTTTGAGACTCTCTCGCTC

Neph3 5' -1072-FW	-1072 to -1053	GACACGCGTCTGTCACCCTCTTCCAAGTG

Neph3 5' -536-FW	-536 to -518	GACACGCGTGACGTGCTGTAGTTTGCAG

Neph3 5' -366-FW	-366 to -352	GACACGCGTGGAAACTGGCGAGGC

Neph3 5' -162-FW	-162 to -145	GACACGCGTCAGACCCCAATTGAGCTG

Neph3 5' -105-FW	-105 to -89	GACACGCGTCATTCATCCGCGTCTCA

*Primers for site-directed mutagenesis *^*b*^		

Neph3 5'-118/NF-κB mutation	-96 to -62	GCGTCTCAGCCGC**TTA**AGTTTCTCAACGGGAAGAG

Neph3 5'-118/Sp1 mutation	-77 to -47	TTCTCAACGGGAAGAG**AAT**GGAGCTCCCGGG

*Primers for ChIP*		

Neph3-5' -81-FW ChIP	-81 to -62	GAGTTTCTCAACGGGAAGAG

Neph3-5' +50-RV ChIP	+50 to +32	GCCTCTGACGCTCTGAAAC

^a^Positions in genomic sequence [GenBank: AC002133] related to Neph3 transcription start site.

^b^For oligonucleotides used in mutagenesis, only the sense primer is shown. The core element is underlined and the mutated oligonucleotides are shown in bold.

### Construction of reporter plasmids

Reporter construct Neph3 5' -5070-Luc was produced by PCR amplification from the TOPO construct using Neph3 5' -5070 as sense primer and Neph3 5' +48 as antisense primer (Table 1). The PCR fragment was further subcloned into a pGL3-Basic vector (Invitrogen) upstream of the luciferase reporter gene using MluI and BglII restriction sites. Deletion constructs with variable lengths of the Neph3 5'-flanking sequence (Figure 2) were generated similarly by PCR amplification from the Neph3 5' -5070-Luc construct. Neph3 5' +48 was used as antisense primer, respective sense primers are shown in Table 1. The construct Neph3 5' -57 was generated by digestion of the Neph3 5' -536 construct with SacI followed by re-ligation. The integrity of all the constructs was verified by sequencing.

### Site-directed mutagenesis

Mutation of the Sp1 and NF-κB binding sites was performed by introducing the point mutations into Neph3 5' -105 plasmid using the QuikChange™site-directed mutagenesis kit (Stratagene, La Jolla, CA, USA), according to the manufacturer's instructions. Oligonucleotides with site-specific mutations are listed in Table 1. The mutations were confirmed by sequencing. The Sp1 and NF-κB mutations were designed based on the previous works by Zhang et al. and Ritchie et al. [44,45], respectively.

### Cell culture

A293 (human embryonic kidney cells) [46] were grown in RPMI 1640 medium (Sigma, St Louis, MO, USA) supplemented with 10% fetal bovine serum (Sigma), 2 mM GlutaMAX (Invitrogen), 100 U/ml penicillin and 100 μg/ml streptomycin (Sigma). Cells were maintained at 37°C in a 5% CO_2 _humidified incubator. *Drosophila *Schneider 2 (S2) cells, a gift from Prof. Jussi Taipale (University of Helsinki, Finland), were grown in Express Five SFM (Invitrogen) supplemented with 10% fetal bovine serum (Sigma), 2 mM GlutaMAX (Invitrogen), 100 U/ml penicillin and 100 μg/ml streptomycin (Sigma) at 25°C without CO_2_.

Human conditionally immortalized podocyte cell line has been previously described and characterized in detail [47]. Briefly, the cell line contains a transgene encoding a temperature-sensitive mutant of SV-40 T antigen. Additionally, the cell line has been stably transfected with the catalytic domain of the human telomerase gene [48]. Cells proliferate at 33°C, where the SV-40 T antigen is active, but when cells are moved to 37°C, the antigen is inactivated, cells stop to proliferate and adopt the phenotype of differentiated podocytes. Experiments were performed on podocytes thermo-switched to 37°C for 14 days using passage numbers between 5 and 20. Cells were cultured in RPMI 1640 medium containing L-glutamine (Sigma), supplemented with 10% fetal bovine serum and insulin, transferrin and selenite (Sigma).

### Transient transfections and luciferase assay

A293 cells were seeded on 24-well plates on preceding day of transfections. Cells were transiently transfected using FuGENE6 (Roche, Basel, Switzerland) with 400 ng of the Neph3 reporter plasmids together with 5 ng of phRL-TK plasmid (Promega, Madison, WI, USA) as an internal control to correct the transfection efficiency. pGL3-Basic (Promega) plasmid was used as a negative control. Cells were lysed 24 h after transfection and luciferase activities were measured using the Dual-Luciferase Reporter Assay System (Promega) according to the manufacturer's protocol. The studied luciferase activities were normalized by luciferase activities produced by co-transfected phRL-TK control plasmid.

### Overexpression of NF-κB and Sp1

NF-κB overexpression studies were performed in A293 cells. In reporter gene assays, cells were transfected and luciferase activities were measured as above. For co-transfection of the Neph3 promoter plasmid along with p50 or/and p65 NF-κB (NF-κB family members p50 and p65), 50 ng of the expression plasmids pcDNA3.1/p50 and pcDNA3.1/p65 (kindly provided by Alan Krensky, Stanford University School of Medicine) were used. The corresponding empty vector pcDNA3.1 was used as a control. The amount of transfected DNA was maintained constant with empty pcDNA3.1 vector.

To investigate the effects of NF-κB p50 and p65 subunits on the endogenous Neph3 RNA level, A293 cells were seeded on 12-well plates and transiently transfected using Lipofectamine™ 2000 (Invitrogen) with 1.5 μg of p50 or/and p65 NF-κB expression plasmids. RNA was extracted 48 h after transfection.

Sp1 overexpression studies were done in *Drosophila *S2 cells lacking endogenous Sp1. Cells were seeded on 12-well plates and on the following day transiently co-transfected using FuGENE6 (Roche) with 500 ng of Neph3 promoter plasmid and 50 ng of pPAC-Sp1 that expresses human Sp1 driven by the *Drosophila melanogaster *actin promoter (a gift from Prof. Dr. Guntram Suske, Philipps-Universität Marburg). The corresponding empty vector pPAC was used as a negative control. Luciferase activities were measured 48 h after transfections and normalized by the protein concentration measured using a Bradford protein assay (Bio-Rad).

### Chromatin immunoprecipitation

The chromatin immunoprecipitation assay was performed using the ChIP-IT™ Express (Active Motif, Carlsbad, CA, USA) according to the manufacturer's instructions with some modifications. Briefly, differentiated human podocytes were cross-linked with 1% formaldehyde for 10 min at room temperature. Cells were washed with ice-cold PBS and the fixation reaction was stopped by adding 0.125 M glycine for 5 min at room temperature. Cells were washed again with ice-cold PBS and scraped from the dish. Cells were pelleted by centrifugation and resuspended in the lysis buffer. After centrifugation, pelleted nuclei were resuspended in the shearing buffer, incubated on ice for 30 min and the chromatin was sheared by sonication (Hielscher Ultrasonics GmbH, Teltow, Germany) at 25% power 5 pulses of 20 sec each on ice into fragments of approximately 200–600 bp. The sheared chromatin was then centrifuged and the supernatant was collected. For immunoprecipitations, 60 μl of chromatin was incubated with 1 μg of Sp1 (Santa Cruz Biotechnology, Santa Cruz, CA, USA), NF-κB p65 (Abcam, Cambridge, UK) or NF-κB p50 (Abcam) antibodies or with rabbit IgG (Zymed Laboratories, South San Francisco, CA, USA), as a negative control, overnight at 4°C with gentle rotation. Immunocomplexes bound to magnetic beads were collected using a magnetic stand, washed extensively, and the protein/DNA crosslinks were reversed and DNA eluted for real-time PCR analysis.

PCR mixtures contained 10 μl of the 2× Power SYBR^® ^Green PCR Master Mix (Applied Biosystems, Foster City, CA, USA), 100 nM of each primer (Table 1) and 5 μl of DNA in a total volume of 20 μl. The following PCR profile in iCycler system (Bio-Rad) was used: preincubation at 95°C for 10 min, 40 cycles of 95°C for 30 s, 55°C for 30 s and 72°C for 30 s. The specificity of the PCR-products was confirmed with melting curve analysis and by size as determined by agarose gel electrophoresis.

### RNA preparation, conventional and quantitative reverse transcription-PCR

Total RNA was isolated from human A293 embryonic kidney cell line (non-transfected and transfected with NF-κB p50 and p65 subunits) and differentiated human podocytes with RNeasy Mini Kit (Qiagen, Hilden, Germany) according to the manufacturer's instruction. RNA samples were then treated with DNase I (Promega). 1 ug of each DNase treated RNA sample was reverse transcribed into cDNA using Primer random p(dN)6 (Roche) and M-MLV Reverse Transcriptase (reverse transcriptase positive (RT+) sample; Promega) as suggested in the manufacturer's instructions. To confirm the RNA origin of the PCR signals, samples that were not reverse transcribed (RT- samples) were analyzed in parallel with the RT+ samples.

To detect endogenous Neph3 mRNA in A293 cells and differentiated human podocytes, the conventional PCR reaction was performed with HotStar Taq DNA polymerase (Qiagen) in a total volume of 20 μl with 400 nM of each primer (Table 1) and 1 μl of cDNA. The PCR was performed as follows: preincubation at 95°C for 15 min, 40 cycles of 95°C for 30 s, 54°C for 30 s and 72°C for 30 s, and final extension at 72°C for 5 min. PCR products were visualized on a 1.5% agarose gel and the identity of the amplification products of the RT+ samples was confirmed by sequencing.

To determine the effect of NF-κB on Neph3 gene expression, the quantitative reverse transcription-PCR analysis was performed in iCycler system (Bio-Rad) using TaqMan chemistry. Commercially available pre-developed TaqMan gene expression assays (Applied Biosystems) for human Neph3 (Assay ID: Hs00375638_m1) and human GAPDH (Hs99999905_m1) were used in the the PCR reaction in a total volume of 20 μl together with TaqMan Fast Universal PCR Master Mix (Applied Biosystems) and 1 μl of cDNA. The following PCR profile was used: preincubation at 95°C for 20 s, 40 cycles of 95°C for 3 s and 60°C for 30 s. Measurements were performed in triplicate. The expression levels of Neph3 RNA were normalized to GAPDH using the comparative C_t _method (ΔΔC_t_).

### Bioinformatics and statistics

MatInspector software using Genomatix matrixes  was exploited to study the putative transcription factor binding sites in the Neph3 5' promoter. The core similarity of 1.0 and matrix similarity of 0.8 were selected to scan sequences for matches.

Statistical analyses were performed using independent samples *t*-test. P values of 0.05 or less were considered to be statistically significant.

## Authors' contributions

MR carried out all the in vitro experiments, drafted the manuscript, participated in the design of the study and computational analysis. SA designed the study, characterized the promoter by computer analysis, assisted in introduction of the in vitro methods, and helped to draft the manuscript. MAS, PWM and GIW created the human podocyte cell line and assisted in the podocyte cell culture. SL and HH participated in the design of the study and helped to draft the manuscript. All authors approved the final manuscript.
